# Maleic and Methacrylic Homopolymers with Pendant Dibutylamine or Dibutylamine Oxide Groups as Kinetic Hydrate Inhibitors

**DOI:** 10.1021/acsomega.2c05713

**Published:** 2022-11-09

**Authors:** Malcolm A. Kelland, Janronel Pomicpic, Radhakanta Ghosh

**Affiliations:** Department of Chemistry, Bioscience and Environmental Engineering, Faculty of Science and Technology, University of Stavanger, N-4036 Stavanger, Norway

## Abstract

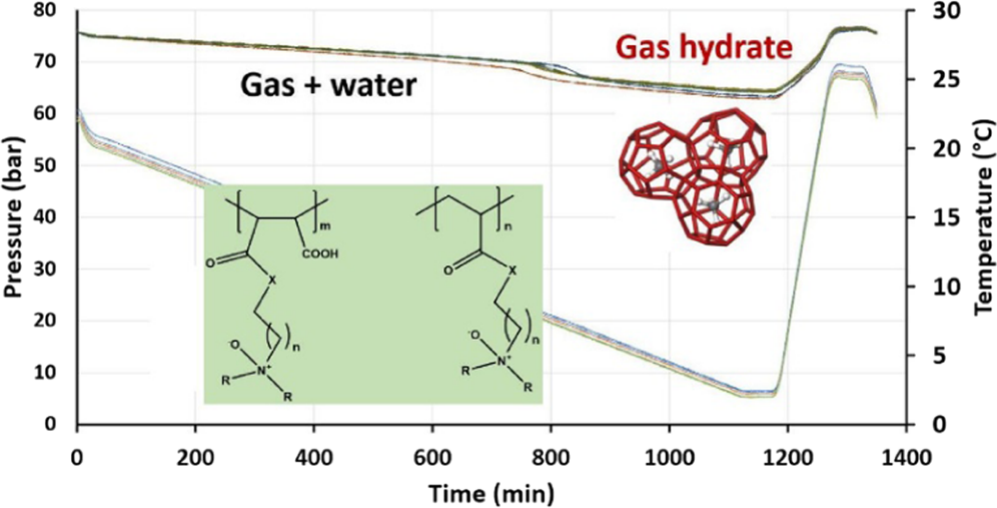

Kinetic hydrate inhibitors (KHIs) are applied in oil and gas fields to prevent gas hydrate formation, most often in cold subsea flow lines. The main component in industrial KHI formulations is a water-soluble polymer with many amphiphilic groups of which the hydrophilic part is most commonly the amide functional group. In the last decade, we have investigated polyamine oxides as alternatives to polyamides due to the strong hydrogen bonding of the amine oxide group. Here, we report the KHI performance of maleic and methacrylic homopolymers with dialkylamine and dialkylamine oxide pendant groups. Performance screening experiments were conducted under high pressure with a Structure II-forming natural gas mixture in steel rocking cells using the slow (1 °C/h) constant cooling test method. Polymers with dibutylamine groups gave much better KHI performance than polymers with dimethylamine or diethylamine groups. Polyamines formed from polymaleic anhydride reacted with 3-(dibutylamino)-1-propylamine (DBAPA) or 2-(dibutylamino)-ethanol (DBAE) gave good water solubility and good KHI performance, probably due to self-ionization between the dibutylamino and carboxylic acid groups. The lack of self-ionization for the methacryl homopolymers of DBAPA and DBAE explains why these polymers are not water-soluble. Oxidation of the maleic or methacryl polyamines to polyamine oxides gave water-soluble polymers with good compatibility with brines (0.5–7.0 wt % NaCl), but only the DBAPA-based polyamine oxides gave improved KHI performance compared to the polyamines. Poly(3-(dibutylamino oxide)-1-propyl methacrylamide) gave a similar performance to commercial *N*-vinyl pyrrolidone:*N*-vinyl caprolactam 1:1 copolymer and without a cloud point in deionized water up to +95 °C.

## Introduction

**Figure 1:**
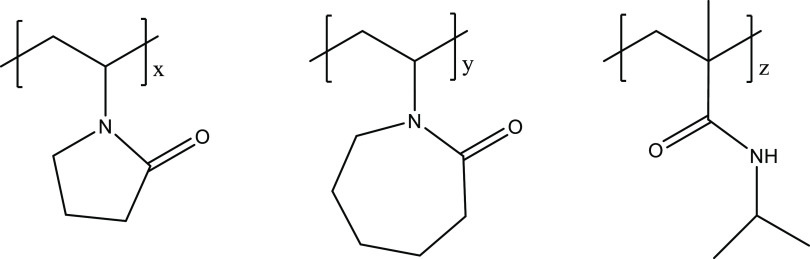
Industrial deployed KHI polymers. Left to right: poly(*N*-vinyl pyrrolidone), poly(*N*-vinyl caprolactam), and poly(*N*-isopropylmethacrylamide).

Kinetic hydrate inhibitors (KHIs) are a class of low-dosage hydrate inhibitors (LDHIs) used to prevent gas hydrate blockages in oil and gas production flow lines, both subsea and on land.^1−9^ The main active ingredients in KHIs are water-soluble polymers mixed with synergists some of which may be the solvent. KHIs delay the gas hydrate formation processes, both at the nucleation and crystal growth stages. The delay time is dependent on many factors, but the thermodynamic driving force (chemical potential), simplified to the subcooling of the system, is the most important factor.^10,11^ The driving force of the system is often described in terms of the subcooling, but other factors including the absolute pressure must be taken into account. There is evidence that KHIs can give total inhibition for an indefinite period up to a certain driving force.^12^ KHI formulated liquids are injected into the produced well stream such that the active polymer content in the produced aqueous fluid is usually less than 1.0 wt %. The vast majority of commercial KHI polymers are amide-based polymers, such as poly (*N*-vinyl pyrrolidone) (PVP), poly (*N*-vinyl caprolactam) (PVCap), poly(*N*-*iso*-propyl methacrylamide) (PNIPMAM), hyperbranched polyesteramides, and copolymers thereof (Figure 1).^13^ More effective and cheaper KHIs are goals to help improve the range of gas hydrate control treatments.

**Figure 2:**
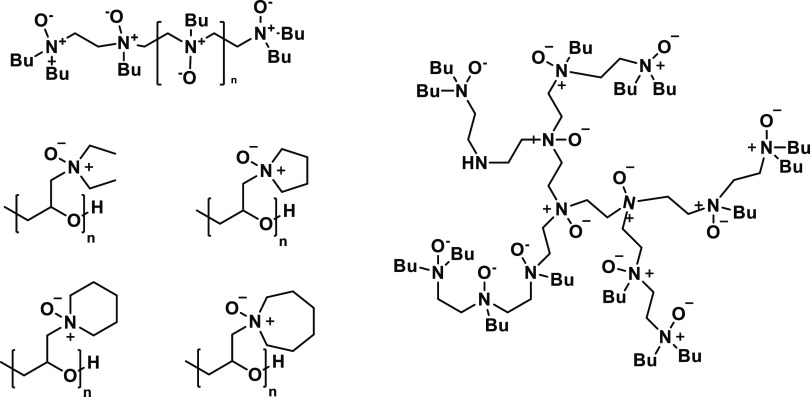
Linear polyethyleneamine oxides (top left), hyperbranched polyethyleneamine oxides (right), and cyclic polyetheramines (middle and bottom left).

Amine oxide is an alternative functional to the amide group in KHI polymers, which we have been exploring for some years. Some of these polyamine oxide classes give good performance with the correct size hydrophobic groups (Figure 2).^14−16^

**Figure 3:**
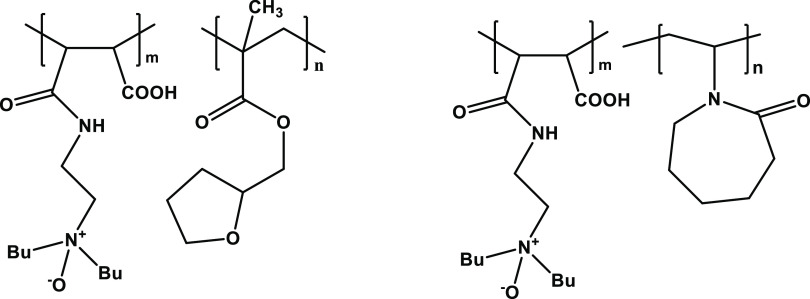
Copolymers formed from the reaction of DBAPA with copolymers of maleic anhydride with tetrahydrofurfurylmethacrylate and *N*-vinyl caprolactam.

We have also recently investigated maleic-based amide polymers as KHIs. The first generation of these KHI polymers relied on small alkylamide substituents such as isobutyl for their performance.^17^ More recent work has led to copolymers with improved performance and excellent compatibility at high temperatures.^18^ We also explored ways to improve the performance of maleic-based copolymers by introducing amine oxide groups. Two examples of polymers with good KHI performance are given in Figure 3 that contain the amine oxide from the reaction of maleic anhydride monomer units with 3-(dibutylamino)-1-propylamine (DBAPA).^19,20^

**Figure 4:**
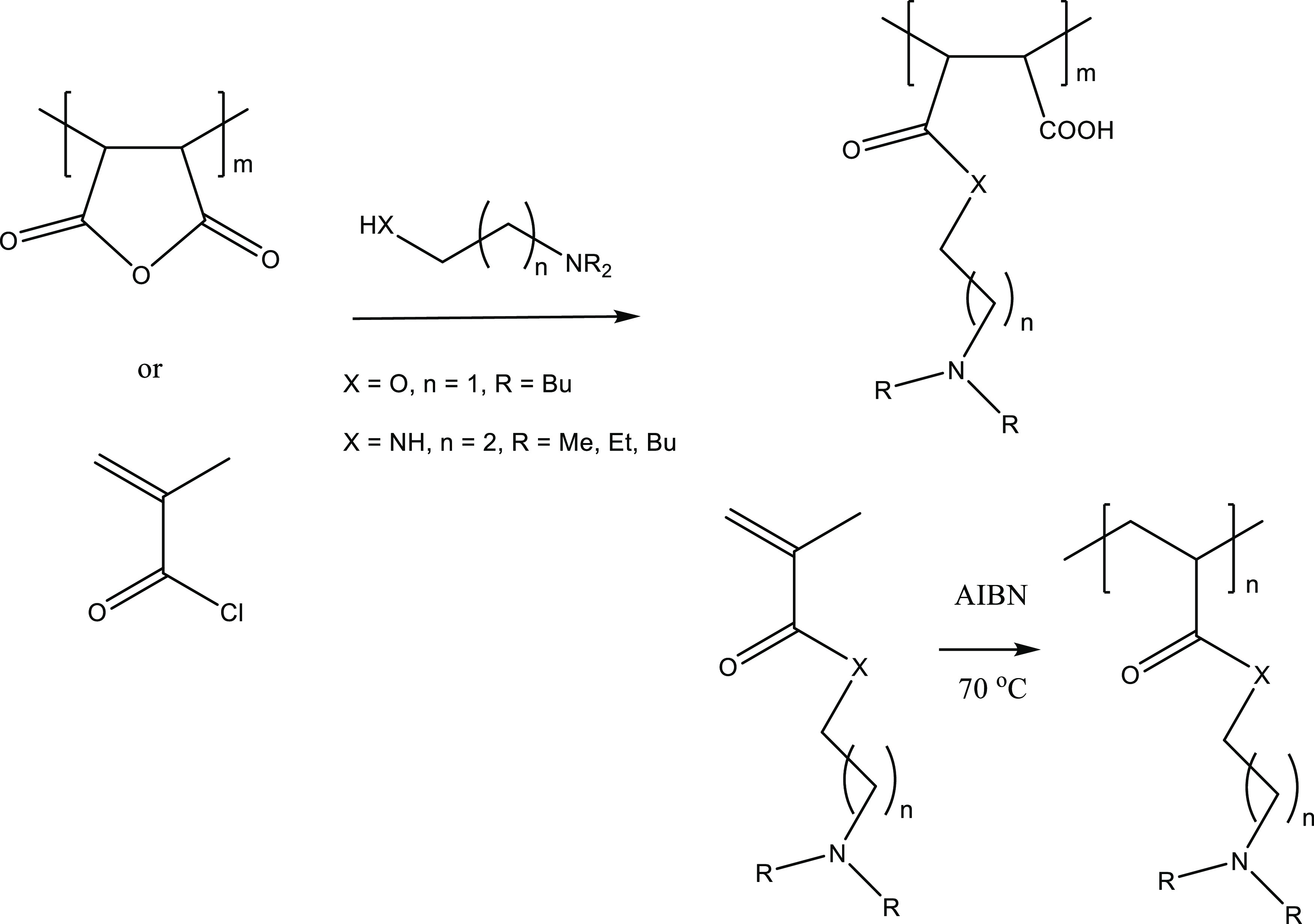
Formation of polyamines with dialkylamino side groups from maleic anhydride or methacryloyl chloride.

DBAPA proved to be a very useful amine to react with maleic anhydride polymers, giving pendant groups that are easily transformed into dibutylamine oxide groups via treatment with hydrogen peroxide. However, we did not explore the amine oxide homopolymers using polymaleic anhydride (PMA). Here we report, the synthesis and KHI performance of these homopolymers as well as the related polymaleic ester amine oxides from the reaction of PMA with 2-(dibutylamino)-ethanol (DBAE). (Figure 4). Further, we also report the polymethacrylamide and polymethacryl esters of DBAPA and DBAE, respectively, and their amine oxide derivatives.

## Experimental Section

### Materials

Maleic anhydride (≥99%, Merck), methacryloyl chloride (97%, Sigma-Aldrich), 3-(dibutylamino)-1-propylamine (DBAPA, 98% Sigma-Aldrich), 2-(dibutylamino)-ethanol (DBAE, 99% Sigma-Aldrich), *o*-xylene (99%, VWR), 2,2′-azobis(isobutyronitrile) (AIBN, 98%, Sigma-Aldrich), 2-butoxyethanol (*n*-BGE, 99%, Acros Organics), 1,2-dimethoxyethane (DME, 99%, VWR), and 2-propanol (iPrOH, 99%, VWR) were used as received. CDCl_3_ was purchased from Cambridge Isotope Laboratories, Inc. Synthesis of polymaleic anhydride (PMA, *M*_w_ = 800, dispersity 3.8) was carried out according to the literature, except that toluene was replaced by *o*-xylene.^19^ Poly(*N*-vinyl caprolactam) (PVCap) (MW approximately 2–4 kg/mol) was supplied from BASF as Luvicap EG, a 41.1 wt % solution of the polymer in monoethyleneglycol (MEG). MEG was removed for this study by repeated precipitation of the polymer from aqueous solution above the cloud and deposition point (ca. 40 °C). *N*-Vinyl pyrrolidone:*N*-vinyl caprolactam 1:1 copolymer (VP:VCap) was also supplied by BASF as Luvicap 55W, as a 53.8 wt % solution in water (MW approximately 2–4 kg/mol), and used as received.

Nuclear magnetic resonance (NMR) spectra were recorded on a Bruker Ascend NMR 400 MHz spectrometer at ambient temperature unless otherwise stated. Gel permeation chromatography/size exclusion chromatography (GPC/SEC) analysis was carried out conducted to determine the polymer molecular weight as well as the polydispersity index (PDI). The apparatus used was a JASCO Chem NAV size exclusion chromatography system. This system was equipped with a PU-2080 Intelligent HPLC pump, an AS-2055 intelligent autosampler, a CO-2065 Intelligent column oven, an RI-2031 Intelligent RI detector, and two commercial columns (TSKgel SuperH4000 and TSKgel GMHXL). PMA molecular weight analysis was done at 40 °C with dimethylformamide (DMF) as eluent and polystyrene standards for calibration. For the methacryl polymers, tetrahydrofuran (THF) was used as solvent at 40 °C, with superH3000 and GMH columns of Tosoh company, Japan, and polymethyl methacrylate standards.

### Synthesis of Maleamide Polymers

PMA, amine (DBAPA, 1 molar equivalent of the maleic anhydride monomer units), and solvent *n*-butyl glycol ether (*n*BGE) were loaded into a vial. The mixture was stirred at 21 °C overnight. PMA, amine (DBAE, 1 equivalent of the maleic anhydride monomer units), and solvent (DME) were loaded into an airtight vial, and the mixture was stirred at 60 °C overnight. ^1^H NMR spectroscopic analysis after the reaction of amine (DBAPA or DBAE) showed no free amine, suggesting a quantitative yield. The maleamide polymers (PMA–DBAPA, PMA–DBAE) were kept in the respective solvent carrier at a determined concentration (25.78%) for KHI testing. Molecular weight data are given in Table 1.

**Table 1:**
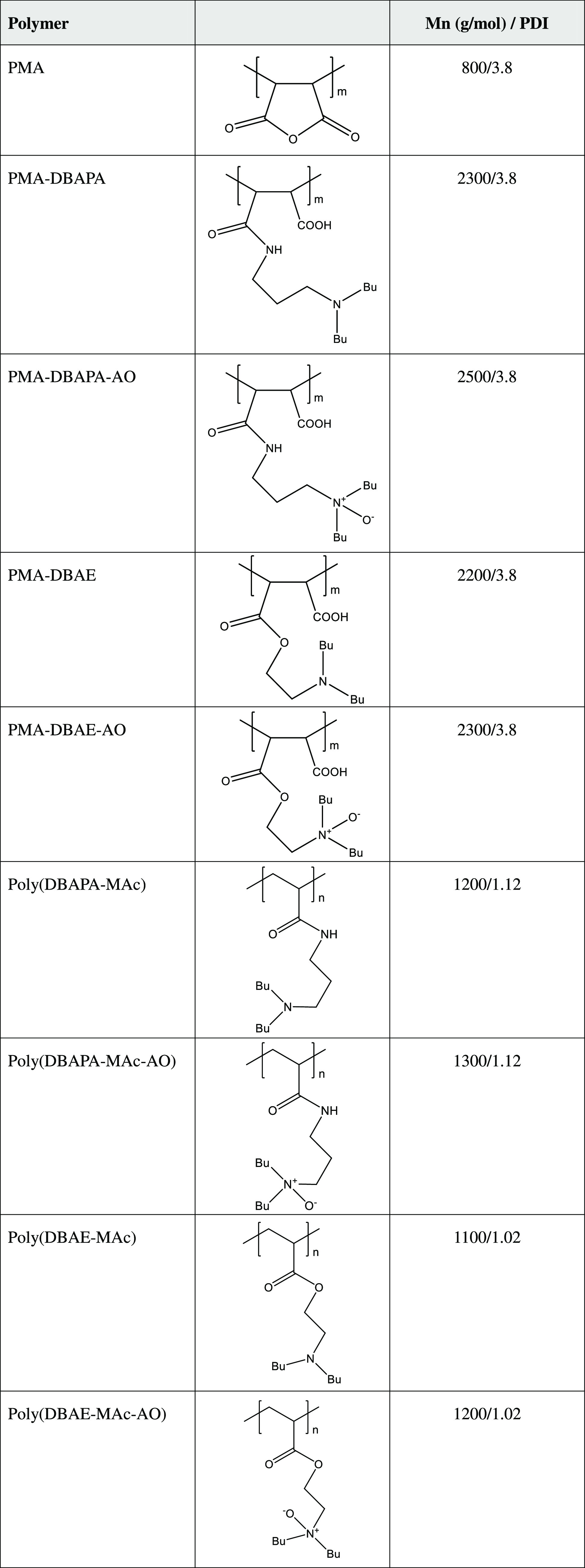
Polymer Molecular Weights and PDI Values Determined by GPC and Calculations for Amine and Amine Oxide Derivatives^a^

a *M*_n_ values for PMA derivatives are calculated knowing the *M*_n_ value for PMA.

### Syntheses of Methacrylate or Methacrylamide Monomers

2-(Dibutylamino)ethyl methacrylate (DBAE-MAc) and 3-(dibutylamino)-1-propyl methacrylamide (DBAPA-MAc) were synthesized following a similar method. DBAPA or DBAE (0.1 mol) and triethylamine (0.1 mol) were dissolved in 100 mL of anhydrous dichloromethane, and the solution was then cooled to 0 °C by an ice bath. Methacryloyl chloride (0.1 mol) was then added dropwise to the solution over 30 min under stirring. The reaction mixture was allowed to warm up slowly to room temperature and kept stirring overnight. After 18 h, the resulting salt was filtered and then the filtrate was washed with 1% hydrochloric acid, saturated sodium bicarbonate solution, brine solution, and finally with distilled water. Then, the organic layer was passed through anhydrous sodium sulfate to remove any water and the organic solvent was removed under reduced pressure. The product was again purified by silica column chromatography using a solvent mixture of hexane:ethyl acetate (9:1). After the solvent evaporation, a liquid was obtained as the final product (Yield: ∼55%).

**Figure 5:**
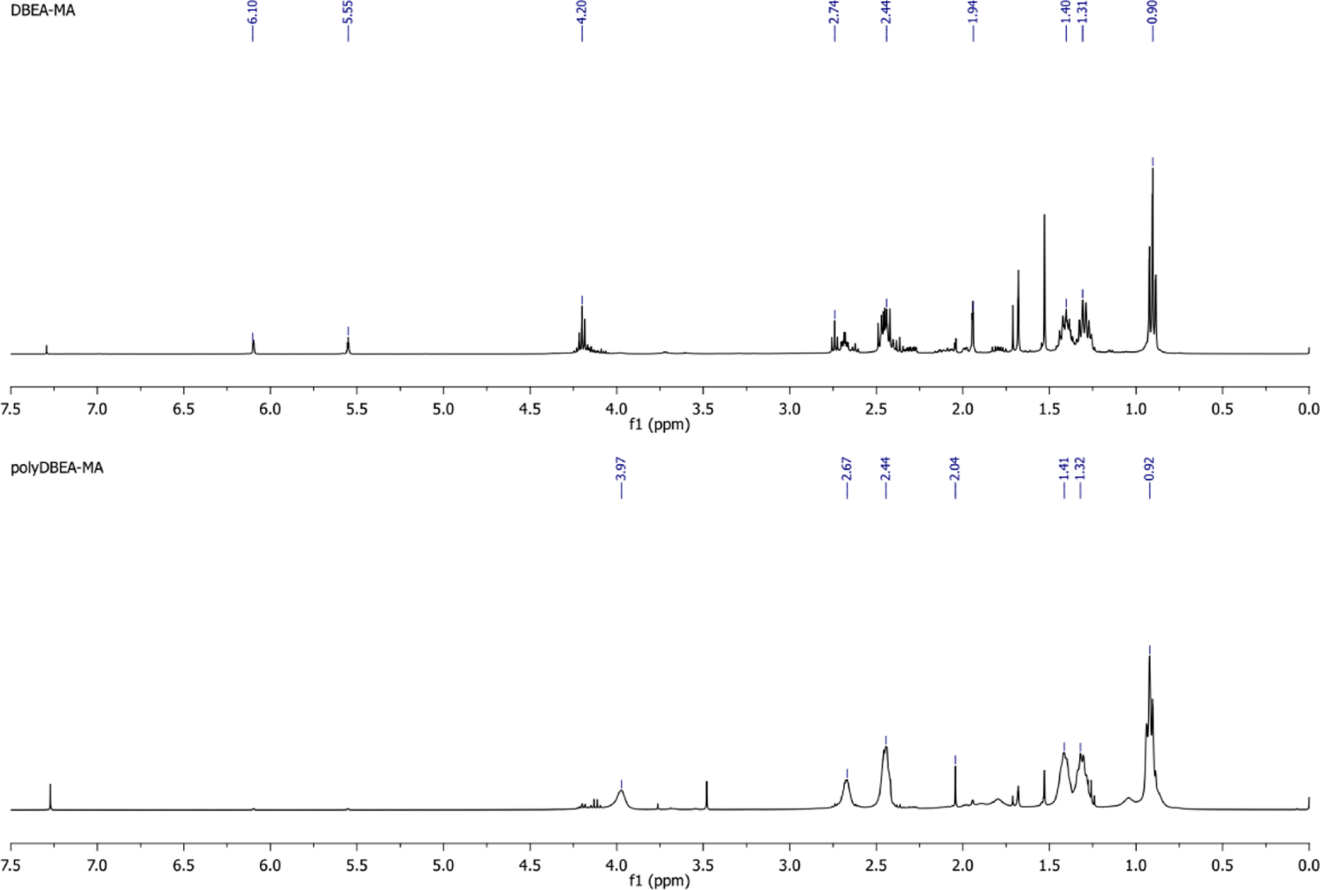
^1^H NMR spectrum of DBAE-MAc (top) and poly(DBAE-MAc) homopolymer (bottom) in CDCl_3_.

^1^H NMR of DBAE-MAc (TMS, CDCl_3_, ppm): 6.1 (1H, C**H**H = C(CH_3_)-), 5.55 (1H, CH**H** = C(CH_3_)-), 4.2 (2H, -OC**H**_**2**_CH_2_N-), 2.74 (2H, -OCH_2_C**H**_**2**_N-), 2.44 (4H, -N(C**H**_**2**_CH_2_CH_2_CH_3_)_2_), 1.94 (3H, CH_2_ = C(C**H**_**3**_)-), 1.40 (4H, -N(CH_2_C**H**_**2**_CH_2_CH_3_)_2_), 1.31 (4H, -N(CH_2_CH_2_C**H**_**2**_CH_3_)_2_), 0.9 (6H, -N(CH_2_CH_2_CH_2_C**H**_**3**_)_2_) (Figure 5).

**Figure 6:**
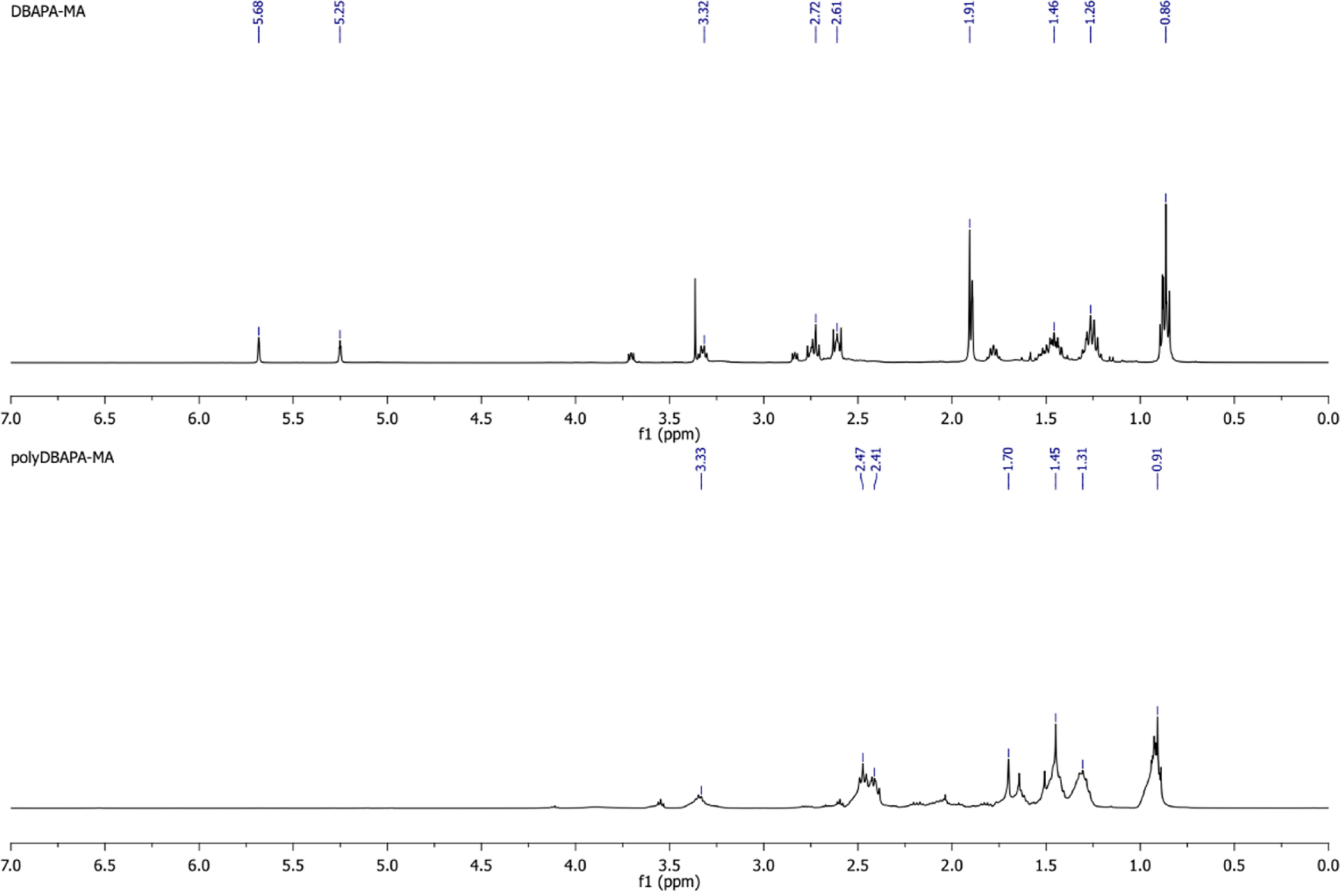
^1^H NMR spectrum of DBAPA-MAc (top) and poly(DBAPA-MAc) homopolymer (bottom) in CDCl_3_.

^1^H NMR of DBAPA-MAc (TMS, CDCl_3_, ppm): 5.68 (1H, C**H**H = C(CH_3_)-), 5.25 (1H, CH**H** = C(CH_3_)-), 3.32 (2H, -NC**H**_**2**_CH_2_N-), 2.72 (2H, -NCH_2_C**H**_**2**_N-), 2.61 (4H, -N(C**H**_**2**_CH_2_CH_2_CH_3_)_2_), 1.91 (3H, CH_2_ = C(C**H**_**3**_)-), 1.46 (4H, -N(CH_2_C**H**_**2**_CH_2_CH_3_)_2_), 1.26 (4H, -N(CH_2_CH_2_C**H**_**2**_CH_3_)_2_), 0.86 (6H, -N(CH_2_CH_2_CH_2_C**H**_**3**_)_2_) (Figure 6).

### Synthesis of Poly(DBAE-MAc) and Poly(DBAPA-MAc)

In a typical procedure, 1 g of DBAE-MAc or DBAPA-MAc and 2 wt % AIBN were first added to a 25 mL Schlenk flask (dried under oven prior to use) sealed with a rubber septum for degassing and kept under N_2_. Next, dry isopropanol (12.7 mL) was charged via a gastight syringe. The flask was degassed and purged with N_2_ three times followed by immersing the flask into an oil bath set at 80 °C. The polymerization lasted 21 h, and it was terminated by removing the organic solvent under reduced pressure. DBAPA-MAc does not polymerize in the presence of solvent following this protocol, whereas DBAE-MAc does. But DBAPA-MAc does polymerize following the same protocol without any solvent. Molecular weight data are given in Table 1.

^1^H NMR of poly(DBAE-MAc) (TMS, CDCl_3_, ppm): 3.97 (2H, -OC**H**_**2**_CH_2_N-), 2.67 (2H, -OCH_2_C**H**_**2**_N-), 2.44 (4H, -N(C**H**_**2**_CH_2_CH_2_CH_3_)_2_), 2.04 (3H, CH_2_ = C(C**H**_**3**_)-), 1.41 (4H, -N(CH_2_C**H**_**2**_CH_2_CH_3_)_2_), 1.32 (4H, -N(CH_2_CH_2_C**H**_**2**_CH_3_)_2_), 0.92 (6H, -N(CH_2_CH_2_CH_2_C**H**_**3**_)_2_) (Figure 5).

^1^H NMR of poly(DBAPA-MAc) (TMS, CDCl_3_, ppm): 3.33 (2H, -NC**H**_**2**_CH_2_N-), 2.47 (2H, -NCH_2_C**H**_**2**_N-), 2.41 (4H, -N(C**H**_**2**_CH_2_CH_2_CH_3_)_2_), 1.70 (3H, CH_2_ = C(C**H**_**3**_)-), 1.45 (4H, -N(CH_2_C**H**_**2**_CH_2_CH_3_)_2_), 1.31 (4H, -N(CH_2_CH_2_C**H**_**2**_CH_3_)_2_), 0.91 (6H, -N(CH_2_CH_2_CH_2_C**H**_**3**_)_2_). (Figure 6).

### Synthesis of Amine Oxide Derivatives of Maleamide and Methacryl Polymers (−NBu_2_ → -NBu_2_^+^O^–^)

PMA–DBAE (25.78 wt % in DME), PMA–DBAPA (25.78 wt % in *n*-BGE), poly(DBAE-MAc) (25.78 wt % in *n*-BGE), and poly(DBAPA-MAc) (25.78 wt % in *n*-BGE) were reacted with 30 wt % solution of hydrogen peroxide. The solution was then stirred for 18 h at room temperature to produce respective amine oxide derivatives, named as PMA–DBAE-AO, PMA–DBAPA-AO, poly(DBAE-MAc)-AO, and poly(DBAPA-MAc)-AO, and kept in the respective solvent carrier at a determined concentration (13.13 wt %) for KHI testing. Calculated molecular weight data are given in Table 1.

### Cloud Point (*T*_Cl_) Measurements

A 2500 ppm solution of the polymer in deionized water (DIW) or sodium chloride brine (0.5, 3.5, or 7.0 wt %) was made. The clarity of the solution was observed at room temperature (20.5 °C). Then, the solution was heated slowly (approximately 5 °C/min close to the *T*_Cl_ value). The cloud point temperature was taken as the first sign of clouding of the solution. Measurements were repeated for confirming reproducibility.

### Kinetic Hydrate Inhibitor (KHI) Performance Tests

These KHI tests were carried out in five 40 mL steel cells that are rocked under pressure in a water bath. The rig (RC5) for these operations was supplied by PSL Systemtechnik, Germany.^21−23^ A synthetic natural gas (SNG) blend was used as supplied by Yara Praxair, Norway (Table 2). The composition was analyzed to be within ±0.1% of all of the required concentrations. The equilibrium temperature (Teq) for sII gas hydrate at approximately 77 bar of SNG was predicted to be 20.5 °C by PVTSim software, Calsep.^24^

**Table 2:** Composition of the Synthetic Natural Gas (SNG) Mixture

component	mol %
nitrogen	0.11
*n*-butane	0.72
isobutane	1.65
propane	5.00
CO_2_	1.82
ethane	10.3
methane	80.4

To screen the performance of the new polymers, we used the slow constant cooling (SCC) test, which has been used by our group for many years. This enables us to compare the performance of new KHIs to a range of previously tested polymers.^24^ The standard procedure for SCC tests was as follows:

1. About 105 mL of KHI solution with dissolved polymer was prepared at least one day before the KHI performance tests to ensure complete dissolution; 20 mL of the KHI solution was added to each cell.
2. Air was removed from the cells by alternate vacuuming and purging with SNG.
3. Approximately 76 bar of SNG was loaded to each cell at 20.5 °C.
4. The cells were cooled at 1 °C/h while rocking with 20 full swings/min with maximum 40° angle; the pressure and temperature data were recorded constantly.

**Figure 7:**
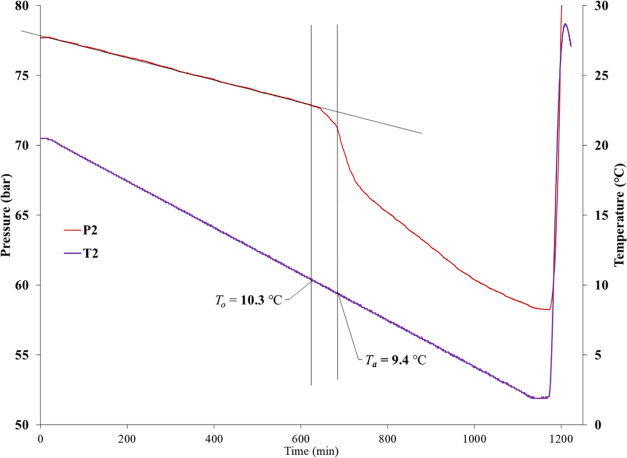
Determination of *T*_o_ and *T*_a_ values for an individual SCC test.

The determination of hydrate onset temperature (*T*_o_) and rapid hydrate formation temperature (*T*_a_) from the temperature and pressure curves obtained from one cell can be seen in Figure 7. In the closed system, the pressure decreased linearly due to the constant cooling of the temperature. Once gas hydrates started to form, the pressure deviated from the original linear track, and this first pressure drop point was marked as *P*_o_. The corresponding temperature at *P*_o_ was determined as *T*_o_. The fastest pressure drop point was marked as *P*_a_, and its corresponding temperature was determined as *T*_a_.

The standard deviation (assuming a normal distribution) for a set of *T*_o_ or *T*_a_ values is no more than 0.6 °C and usually less than 0.3 °C. The scattering still allows for a rough ranking of the performance of the KHI samples as long as sufficient tests are carried out for a statistically significant difference using a *t*-test. Depending on the variation in average *T*_o_ between samples, 5–10 tests suffices in most cases to get a significant difference at the 95% confidence level (*p* < 0.05).^25^

## Results and Discussion

### Maleic-Based Polyamine Oxides Synthesis

Polymaleic anhydride (PMA) was made in *o*-xylene as previously described to give a low-molecular-weight polymer.^19^ As the *o*-xylene forms an end cap, this means the average number of MA monomer units in the polymer is about seven. For amination of PMA at room temperature, we used *n*BGE as a high-flash-point solvent that has previously been shown to have a weak synergetic effect with polymaleamides.^20,21^ It was very important that PMA was kept dry before reacting with amines, otherwise we poorer KHI performance was obtained. This was probably due to the hydrolysis of PMA to polymaleic acid, which is not amidated by reaction with amines at room temperature.

### Polymer Solubility and Cloud Point

**Figure 8:**
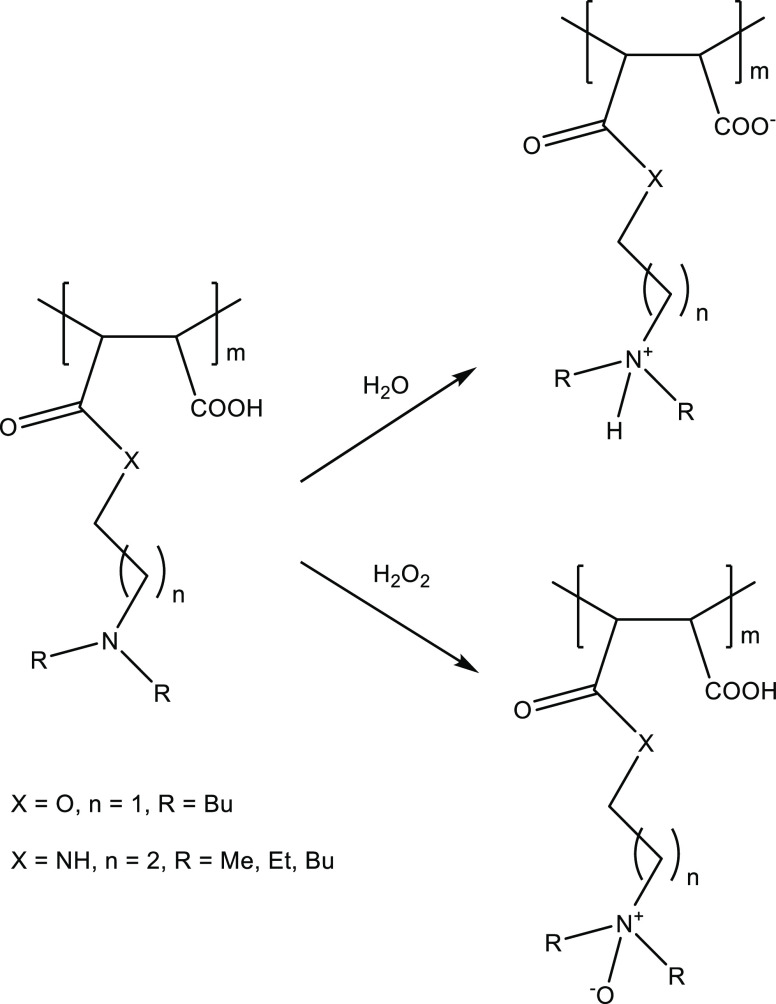
Proposed quaternization (self-ionization) or amine oxide formation of the polymaleic dialkylamino groups.

The solubility data and cloud points for all polymers in deionized water and various sodium chloride (NaCl) brines are summarized in Table 3. Starting with the PMA derivatives, the PMA–DMAPA and PMA–DEtAPA derivatives showed no cloud point at all temperatures even with 7.0% NaCl brine. In contrast, PMA–DBAPA with the larger and more hydrophobic dibutylamino head group meant that an opaque solution was observed in DIW at room temperature. However, over time or on heating, the solution became clear. We attribute this behavior to the self-ionization of the carboxylic acid and dibutylamine groups within the polymer as illustrated in Figure 8. An indirect confirmation comes from poly(DBAPA-MAc), which is insoluble in DIW even after heating because it does not contain acid groups for self-ionization. As expected, the amine oxides of both polymaleic and polymethacryl dibutylamine derivatives gave better solubility than the amines. For example, both poly(DBAPA-MAc) and poly(DBAE-MAc) were found to be insoluble in DIW, but the equivalent amine oxide homopolymers poly(DBAPA-Mac-AO) and poly(DBAE-Mac-AO) dissolved easily in DIW. Both of these amine oxide homopolymers gave decreasing cloud points as the brine concentration increased.

**Table 3:** Cloud Points of 2500 ppm Solutions of Polymer in DIW and Various NaCl Brines^a^

	cloud point (*T*_cl_) (°C)				
polymer	DIW	0.5 wt % NaCl	3.5 wt % NaCl	7.0 wt % NaCl	comments
PMA–DMAPA	>95	>95	>95	>95	
PMA–DEtAPA-AO	>95	>95	>95	>95	
PMA–DBAPA	>95	>95	>95	>95	initially opaque
PMA–DBAPA-AO	>95	>95	>95	>95	
PMA–DBAE	>95	>95	>95	>95	opaque in NaCl brines
PMA–DBAE-AO	>95	>95	39	32	
poly(DBAPA-MAc)	insoluble				heating → opaque solution
poly(DBAPA-MAc- AO)	>95	90	58	55	
poly(DBAE-MAc)	insoluble				heating → opaque solution
poly(DBAE-MAc-AO)	>95	81	43	33	

a Solutions are clear unless otherwise stated.

### KHI Performance Screening Results

The results obtained from slow constant cooling tests at polymer concentration of 2500 ppm (0.25 wt %) are summarized in Table 4. Deionized water (DIW), PVCap, and VP:VCap were also tested for comparison. In general, we use *T*_o_ values to gauge the performance of a KHI as total inhibition of macroscopic hydrate formation is best to avoid any chance of deposits building up in the flow line. The *T*_a_ values in Table 4 for both the maleic and methacryl polymers were found to be all quite close to the *T*_o_ values (<1 °C). This suggests that these polymers do not have a strong effect of preventing macroscopic crystal growth.

**Table 4:** KHI Test Results for 2500 ppm Polymers in DIW

polymer	*T*_o_ (av.) [°C]	SD [°C]	*T*_a_ (av.) [°C]	comments
no additive	17.1	0.5	16.9	
PVCap	9.8	0.4	9.4	
VP:VCap 1:1	7.1	0.3	5.9	
PMAcid	16.1	0.6	15.9	
PMA–DMAPA	14.7	0.5	14.2	
PMA–DEtAPA-AO	14.4	0.4	14.0	
PMA–DBAPA	9.5	0.4	9.4	
PMA–DBAPA-AO	8.7	0.3	8.3	
PMA–DBAE	7.7	0.3	7.4	
PMA–DBAE-AO	10.2	0.3	9.9	
poly(DBAPA-MAc)				insoluble, not tested
poly(DBAPA-MAc- AO)	7.4	0.6	6.8	
poly(DBAE-MAc)				insoluble, not tested
poly(DBAE-MAc- AO)	11.5	0.5	11.3	

We will first discuss the maleic-based homopolymers in Table 4, which are all made from PMA with *M*_n_ = 800 g/mol. This means all polymer derivatives will have an average of eight maleic units. For the dialkylamine derivative, as Table 4 shows, PMA homopolymers with pendant dimethylamino or diethylamino groups (PMA–DMAPA and PMA–DEtAPA-AO, respectively) gave poor KHI performance with average *T*_o_ values of 14.7 and 14.4 °C, respectively. The diethylamino polymer was derivatized to amine oxide, which normally improves the performance (see amine oxide results below), underlining the poor effect of using ethyl groups. However, for PMA–DBAPA, the *T*_o_ value was 9.5 °C similar to PVCap. This shows that the larger butyl groups on the pendant amine give far better KHI effect than methyl or ethyl groups as has been observed previously for many other polymer classes.^13,14,21,24^ (Dipropylaminopropylamine was not available to give a polymer with propyl groups). We also investigated the reaction product of PMA with DBAE as this is a readily available alkanolamine with the desired dibutylamino group, as found in DBAPA. Interestingly, PMA–DBAE performed better than PMA–DBAPA, with an average *T*_o_ value that was 1.8 °C lower (Av. *T*_o_ = 7.7 °C). This is a statistically significant difference. At 2500 ppm, the molar concentration of PMA–DBAE is a little higher than that of PMA–DBAPA, which may partly account for the improved performance. Another possibility is that the chain length of DBAE is shorter than that of DBAPA, which gives a higher density of dibutylamino groups on the polymer surface of PMA–DBAE. If an old sample of PMA was used to make PMA–DBAPA or PMA–DBAE, we obtained poorer KHI performance. For example, PMA–DBAE gave *T*_o_ = 13.1 °C and *T*_a_ = 12.0 °C with an old sample of PMA, which we assume was open to the atmosphere and had partially hydrolyzed.

The dibutylamino end groups in these DBAPA or DBAE-derivatized PMA polymers have the possibility to be protonated, either by the effect of dissolved acid gas CO_2_ or internally via transfer of a proton from a carboxylic acid group (Figure 8). This quaternization of the dialkylamino groups could be taking place in solution and give improved performance as we knew from past studies that polymers with pendant quaternary ammonium groups can have good KHI performance.^26^ However, we also knew that amine oxide groups in polymers can give good KHI performance. One study showed that a series of polyamine oxides were significantly better as a KHI than the corresponding polyamines, as well as giving better water solubility.^16^ Therefore, we synthesized several amine oxides of the maleic polymers by reaction with hydrogen peroxide as previously described.^19,20^ As expected, PMA–DBAPA-AO performed a little better than PMA–DBAE-AO. The result was statistically significant at the 95% confidence level by a t-test. Surprisingly, we found that PMA–DBAE-AO performed significantly worse than PMA–DBAE. The average *T*_o_ value was 2.5 °C higher for the polyamine oxide. We are not sure of the reason for this, but we speculate that internal hydrogen bonding is more favored for PMA–DBAE-AO and this reduces its hydrogen bonding with hydrate particles and the bulk water.

We also investigated the performance of PMA–DBAPA and PMA–DBAPA-AO at varying concentrations. A lower-*M*_w_ PMA with *M*_n_ 400 g/mol and PDI = 9.15 made in mixed xylenes was used. This was because we ran out of the PMA batch that had *M*_w_ 800 g/mol. The lower *M*_w_ of this PMA still gave quite good KHI performance when amidated with DBAPA, even though the average number of monomer units (and dibutylamine oxide groups) is only about four (Table 5). The polyamine oxides gave better KHI performance than the polyamines (as seen for PMA–DBAPA-AO in Table 4) and also improved with increasing polymer concentration.

**Table 5:** KHI Performance of PMA–DBAPA and PMA–DBAPA-AO at Varying Concentration^a^

polymer	concentration (ppm)	Av. *T*_o_	Av. *T*_a_
PMA–DBAPA	1000	11.9	11.7
PMA–DBAPA-AO	1000	9.9	9.8
PMA–DBAPA	2500	10.5	9.8
PMA–DBAPA-AO	2500	9.0	8.7
PMA–DBAPA	5000	10.1	9.2
PMA–DBAPA-AO	5000	8.9	8.1

a PMA *M*_n_ 400 g/mol, PDI = 9.15 used as starting material.

To complement the maleic polymers, we synthesized the equivalent methacryl polymers with pendant dibutylamino groups. DBAPA-MAc has been reported previously as a comonomer with *N*-isopropylmethacrylamide for making KHIs, but the performance of the homopolymer has not been reported.^27^ Other dialkylaminopropylmethacrylamido homopolymers have been reported but not the dibutylamino derivative.^28^ DBAPA-MAc and DBAE-MAc groups monomers did not homopolymerize in iPrOH with AIBN, but they did polymerize in bulk to give gels that could be dissolved in organic solvents such as low alcohols.

The ^1^H NMR spectra for both poly(DBAPA-MAc) and poly(DBAE-MAc) gave broad peaks indicating substantial polymerization. The molecular weights were determined by GPC and were surprisingly low (1200 and 1100 g/mol) respectively with low PDI values (Table 1) given the broadness of the NMR resonances. Repeated GPC measurements in DMF using polystyrene standards instead of the original THF gave slightly lower *M*_n_ values. Neither polyamine was soluble in water at room temperature (20.5 °C). However, both the polyamine oxide derivatives poly(DBAPA-MAc-AO) and poly(DBAE-MAc-AO) were soluble at 2500 ppm in DIW and behaved as thermoresponsive polymers in brines as summarized in Table 3.

The KHI test results of the two polyamine oxides using the SCC method were different. Poly(DBAPA-MAc-AO) gave excellent performance with an average *T*_o_ value of 7.4 °C, similar to that of VP:VCap which has a cloud point of about 85 °C at 2500 ppm in DIW. Poly(DBAE-MAc-AO) gave a poorer KHI performance with an average *T*_o_ value of 11.5 °C. The poorer performance compared to poly(DBAPA-MAc-AO) is probably not due to the polymer molecular weights as they are similar to the GPC data. The structural differences are that the dibutylamine oxide group in poly(DBAE-MAc-AO) is closer to the backbone than for poly(DBAPA-MAc-AO) and is subtended by an ester rather than amide group. It is known that amide is a better hydrogen-bonding group than ester, which may play a role in KHI efficiency. A further clue might come from the maleic polymers. A poorer performance was also observed for the maleic polyamine oxide PMA–DBAE-AO compared to the parent polyamine, PMA–DBAE. In this case, PMA–DBAE has the ability to self-ionize to form pendant dibutylammonium groups as discussed earlier. Thus, both the polymaleic and polyacryl amine oxide derivatives with DBAE performed worse than the corresponding DBAPA polymers. Computer modeling might help unravel this mystery, and we plan to carry this out in the near future.

## Conclusions

A series of maleic and methacrylic homopolymers with dialkylamine and dialkylamine oxide pendant groups have been synthesized and screened for KHI performance using a Structure II-forming gas mixture in steel rocking cells using the slow (1 °C/h) constant cooling test method. Polymers with dibutylamine groups gave much better KHI performance than polymers with dimethylamine or diethylamine groups. The maleic-based polyamines PMA–DBAPA and PMA–DBAE made using 3-(dibutylamino)-1-propylamine (DBAPA) or 2-(dibutylamino)-ethanol (DBAE), respectively, gave good water solubility and good KHI performance, particularly the DBAE derivative. This was probably due to self-ionization between the dibutylamino and carboxylic acid groups giving KHI-active dibutylammonium end groups. The methacryl homopolymers poly(DBAPA-MAc) and poly(DBAE-MAc) cannot self-ionize, which explains why they were not water-soluble. Oxidation of the DBAPA groups in the maleic and methacryl polymers gave PMA–DBAPA-AO and poly(DBAPA-MAc-AO), both of which gave excellent KHI performance, with the latter giving very similar results to VP:VCap 1.1 copolymer. The lower performance of the DBAE-AO-based polyamine oxides, compared to the parent DBAE polyamines, is not currently understood and will be further investigated also by computer simulations. We are also investigating these new polyamine oxide homopolymers at other test conditions, as well as synthesizing new maleic and methacryl copolymers with dibutylamine oxide groups with a view to improving the performance further.
